# Metronidazole-Induced Encephalopathy (MIE)

**DOI:** 10.7759/cureus.33133

**Published:** 2022-12-30

**Authors:** Dinesh M Chaudhari, Pushpendra Nath Renjen, Urusa Hasan, Nidhi Goyal, Kamal Ahmad

**Affiliations:** 1 Internal Medicine/Neurosciences, Indraprastha Apollo Hospital, New Delhi, IND; 2 Neurosciences, Indraprastha Apollo Hospital, New Delhi, IND; 3 Internal Medicine, Indraprastha Apollo Hospital, New Delhi, IND; 4 Radiodiagnosis, Indraprastha Apollo Hospital, New Delhi, IND

**Keywords:** altered sensorium, magnetic resonance imaging, toxicity, dentate, metronidazole

## Abstract

Metronidazole is an antibiotic often used to treat bacterial infections in the vagina, skin, liver, stomach, joints, brain and spinal cord, heart, lungs, or bloodstream. It is an essential drug for treating anaerobic bacterial infections, microaerophilic bacterial infections, and protozoal infections. It is cytotoxic to many facultative anaerobic microorganisms. Metronidazole can be taken by most children and adults but might not be suitable for some people. It may produce different neurologic side effects like cerebellar dysfunction, peripheral neuropathy, vestibulotoxicity, visual impairment, encephalopathy, ataxic gait, seizures, dysarthria, and cochleotoxicity. We report a case of a gentleman in his early 60s with a liver abscess and a history of three weeks of use of metronidazole therapy presenting with altered sensorium, abnormal gait, and slurring of speech. MRI brain showed bilateral symmetrical hyperintensities involving the dentate nuclei of the cerebellum and dorsal brain stem without evidence of any diffusion restriction suggestive of metronidazole-induced encephalopathy (MIE).

## Introduction

Metronidazole is a synthetic 5-nitroimidazole with multiple uses as an anti-protozoal and anti-anaerobic drug. It is an essential drug for anaerobic, microaerophilic, and protozoa infections. It imparts a cytotoxic effect on many facultative anaerobic bacteria [1]. It has been used as the primary treatment of many conditions for the last five decades and is the most preferred drug for managing anaerobic bacterial infections. Metronidazole is a cost-effective drug with minimal adverse effects; it includes nausea, diarrhea, vomiting, and mouth dryness. Neurotoxicity is occasional, primarily in headaches, dizziness, peripheral neuropathy, and vertigo. Cerebellar toxicity is an uncommon but grave central nervous system adverse effects can be seen in magnetic resonance imaging (MRI) in some cases [2].

## Case presentation

Our case is of a male in his early 60s who was previously admitted to our hospital with complaints of high-grade fever, chills, abdominal pain, and vomiting. He was diagnosed with amoebic liver abscess. The patient also had diabetes during his stay in the hospital, and his glucose level was managed with insulin. The patient was discharged later from the hospital with improvement in his symptoms. He was initially treated with IV metronidazole for seven days, followed by oral metronidazole 800 mg TID for the next two weeks. Approximately 10 days later, he presented with altered sensorium, abnormal gait, and slurred speech. There was no history of fever, cough, headache, seizures, or other focal neurological deficits. There was no history of consumption of alcohol. The patient had no history of relevant drug intake or seizures in the past. On examination, the patient was afebrile with a heart rate of 100 beats per min, blood pressure of 110/70 mmHg, and respiratory rate of 14 breaths per min. There was no icterus, pallor, clubbing, cyanosis, lymphadenopathy, or pedal edema. The patient was conscious but irritable, with occasional disorientation. Neurological examination showed bilateral, normally reactive pupils with full external eye movement. He had an ataxic gait with no motor weakness and no signs of meningeal irritation. The patient did not have appendicular ataxia nor did he have nystagmus.

Biochemical screening and relevant laboratory results were all within normal limits (Table 1). The urine toxicology panel (10 drugs panel) was negative. Serum ammonia levels were found to be 31 μg/dL. Thyroid function tests, serum folate, and vitamin B12 levels were within normal limits. Viral markers like hepatitis B surface antigen (HBsAg), hepatitis C tests, and HIV were negative. CSF analysis was normal.

**Table 1:** Laboratory results of the patient. HBsAg: hepatitis B surface antigen

Test	Result
Urine toxicology panel (10 drugs panel)	Negative
Arterial blood gas analysis	Within normal limits
CBC	Normal
Serum electrolytes	Normal
Serum ammonia	31 μg/dL
Thyroid function tests	Normal
serum folate	Normal
Vitamin B12 levels	Normal
HBsAg	Negative
Hepatitis C	Negative
HIV	Negative
CSF analysis	Within normal limits

**Figure 1:**
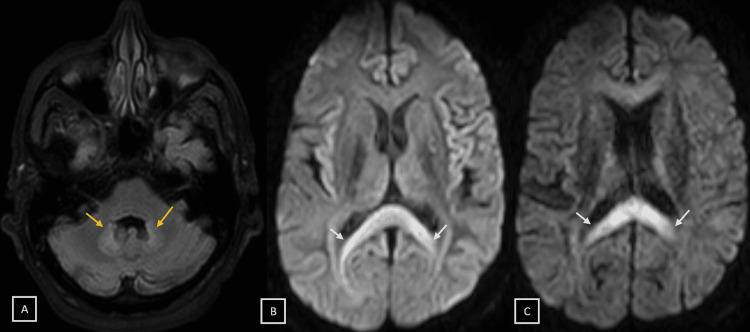
MRI brain of the patient. MRI brain showing bilateral symmetrical hyperintensities involving the dentate nuclei of the cerebellum (yellow arrows) and dorsal brain stem without evidence of any diffusion restriction (A) along with extensive signal alteration and diffusion restriction in the body (B) and genu (C) of corpus callosum (white arrows), suggestive of metronidazole-induced encephalopathy (MIE).

Our patient was evaluated urgently with an MRI brain to rule out any acute ischemic pathology. On presentation, magnetic resonance imaging (MRI) brain revealed extensive uniform altered signals with diffusion restriction in the corpus callosum (genu and body). MRI brain also showed bilateral symmetrical hyperintensities involving the dentate nuclei of the cerebellum and dorsal brain stem without evidence of any diffusion restriction (Figures 1A-1C). 

The findings are representative of metronidazole-induced encephalopathy (MIE); hence the patient was diagnosed with the same, and metronidazole was immediately stopped. After stopping metronidazole therapy, the patient was started on IV fluids and other supportive measures. The patient responded to the treatment and showed significant improvement with supportive treatment, the sensorium improved within three days followed by improvement in gait and speech over the next one week.

## Discussion

Metronidazole (anti-protozoal and anti-microbial) has extensive usage in surgical and medical patients. It contains synthetic 5-nitroimidazole that is dormant on administration and becomes metabolically active on reaching the mark. Metronidazole is a well-tolerated drug with lesser adverse effects. Metallic taste, headache, and nausea are the common side effects. Neurological adverse effects are uncommon; however, mild-to-moderate peripheral neuropathy has been reported widely [3]. When the drug gains access to cerebrospinal fluid (CSF), it may have adverse neurological effects. These range from diffuse encephalopathy to seizures or chronic/acute cerebellar symptoms. Neuroimaging is usually not indicated for such patients, and it is often done to rule out other intracranial pathologies. Imaging is ambiguous in most patients who present with diffuse encephalopathy or seizures [4]. Lately, multiple cases of chronic/acute cerebellar toxicity have been reported [2]. MRI findings like signal alterations in the dentate nucleus are seen in most of the patients in this class. Prompt recognition of imaging abnormalities is important because immediate withdrawal of metronidazole can rapidly reverse signal changes and clinical improvement. The precise pathophysiology of cerebellar toxicity remains unclear [2]. Metronidazole penetrates the blood-brain barrier. It can result in imaging and histological findings that are like Wernicke’s encephalopathy. Neurotoxicity is usually not dependent upon the duration and total dosage administered (oral or intravenous). Central nervous system toxicity induced by metronidazole usually involves the splenium of the corpus callosum, dental nuclei, and the dorsal brainstem. These lesions are symmetrical and bilateral in the majority of the patients. The specific location in metronidazole-induced CNS toxicity includes inferior colliculus and cerebellar dentate nuclei. These lesions appear as fluid-attenuated inversion recovery (FLAIR) and T2 hyperintense areas with no mass effect or contrast enhancement on imaging. Restricted diffusion with variable apparent diffusion coefficient (ADC) values is seen in many patients. The acute nature of cell damage may lead to lower ADC values [5,6]. Complete reversal of imaging findings and symptoms is often the final clinical outcome. Follow-up imaging is not mandatory, particularly after the complete resolution of symptoms [2].

## Conclusions

MIE is a rare but relatively reversible condition with an excellent prognosis. Clinicians must maintain a high index of suspicion for patients on metronidazole who develop any neurological symptoms. A thorough clinical history and MRI scans remain the cornerstone for diagnosis.
